# Bayesian Estimation of the True Seroprevalence and Risk Factor Analysis of Bovine Leukemia Virus Infection in Pakistan

**DOI:** 10.3390/ani11051404

**Published:** 2021-05-14

**Authors:** Ali Sakhawat, Marzena Rola-Łuszczak, Zbigniew Osiński, Nazia Bibi, Jacek Kuźmak

**Affiliations:** 1 National Veterinary Laboratories, Islamabad 45500, Pakistan; sakhawatbalti@yahoo.com; 2 Animal Quarantine Department, Ministry of National Food Security and Research, Peshawar 25000, Pakistan; 3 Department of Bio Sciences, COMSATS University, Islamabad 45500, Pakistan; nazia.bibi@comsats.edu.pk; 4 National Veterinary Research Institute, 24-100 Puławy, Poland; zbigniew.osinski@piwet.pulawy.pl (Z.O.); jkuzmak@piwet.pulawy.pl (J.K.)

**Keywords:** bovine leukemia virus, seroprevalence, risk-factors, Bayesian approach

## Abstract

**Simple Summary:**

The enzootic bovine leucosis is the most common and economically important viral disease of cattle, caused by bovine leukemia virus (BLV). In cattle, infection with BLV leads to decreased milk production and premature culling and also impairs the immune system predisposing animals to other infections and increasing severity of disease. The aim of this study was to estimate the true seropositivity to BLV at the farm and within-farm levels in Pakistan, using a latent class analysis. In addition, some factors influencing BLV seropositivity were analyzed. We tested 1380 dairy cattle from 451 herds and 92 water buffalo. Analysis at the within-herd and herd levels showed 3.8% of cattle and 1.4% of herds were truly seropositive. All 92 serum samples from water buffalo were negative. The study demonstrated strong association between BLV seroprevalence and herd size but not with common housing of cattle representing indigenous breeds with exotic breed or their crossbred and also common housing of cattle and water buffalo.

**Abstract:**

The objective of this study was to determine the true seroprevalence of bovine leukemia virus (BLV) infection in dairy cattle from Pakistan at the animal and herd-level. We tested 1380 dairy cattle from 451 herds and 92 water buffalo. The sera were tested by ELISA and the results were analyzed using Bayesian inference. The median posterior estimate of the herd level true BLV prevalence was 1.4%, with a 95% credible interval (CI) 0.7–3.1, whereas the median posterior estimate of the within-farm true seroprevalence was 3.8% with a 95% CI 2.8–4.8. All 92 sera collected from water buffalo were negative. Several risk factors potentially associated with seropositivity to BLV infections in Pakistan were analyzed using logistic regression model based on calculation of an odds ratio (OR). The study showed an association between seropositivity and medium herd (≥50) size (OR = 23.57, 95% CI: 3.01–103.48). Common housing of indigenous cattle with exotic-breed cattle (OR = 0.67, 95% CI: 06–2.35) or housing indigenous or their crossbred cattle with exotic-breed cattle (OR = 0.95, 95% CI: 0.14–3.01) had no effect on the BLV seroprevalence. Similarly, common housing of cattle and water buffalo was not risk factor for increased BLV seropositivity (OR = 27.10, 95% CI: 0.63–119.34).

## 1. Introduction

Bovine leukemia virus (BLV) is the causative agent of enzootic bovine leukosis (EBL), a neoplastic disease of the lymphatic tissue, which is classified to the genus *Deltaretrovirus* of the *Retroviridae* family [1,2]. In most infected cattle, infection with BLV remains clinically silent but about 30% develop persistent lymphocytosis, characterized by polyclonal expansion of B cells, and approximately 2–5% of predominantly adult cattle, older than 3–5 years, develop lymphoid tumors [3]. The main characteristic of EBL from an epidemiological viewpoint is its very slow spread and clinical manifestation, usually seen 1–8 years after infection [4]. Natural BLV infection has been confirmed in species such as *Bos taurus* (domestic cattle), *Bos indicus* (zebu), *Babalus bubalis* (water buffalo), and *Bos grunniens* (domestic yaks), however, BLV can be transmitted experimentally to other species [5]. There is no evidence to suggest that any significant reservoir of BLV exists among other species, including wildlife ruminants.

BLV is highly cell-associated, and the free virus is rarely or never found in the blood of infected cattle [6]. Thus, BLV transmission can occur horizontally or vertically through the transfer of BLV-infected cells (B-lymphocytes, monocytes and macrophages, epithelial cells) present in the blood and other body fluids [7]. Direct contact between infected and uninfected animals is considered to be the main risk factor for within-herd transmission [8], while transmission between herds is determined by the introduction of new, infected animals [9]. Transfer of infected blood can also occur via hematophagous insects such as horse flies (*Tabanus* spp.) [10], and the presence of flies in a stable has been considered to be a risk factor for higher within-herd prevalence of BLV [11].

BLV infection has a worldwide distribution, and epidemiological studies based on serology showed high prevalence in North and South America (30–80%), while in some Asiatic and Middle Eastern countries, the seroprevalence was lower, i.e., about 20% [2,12]. In countries endemically affected by EBL, the impact of the BLV infection is determined by trade restrictions on breeding animals, premature culling, production losses due to weight and milk loss, and carcass condemnation at slaughter [13,14,15]. Additionally, BLV infection also impairs the immune system and predisposes animals to multiple coinfections, increasing the severity of the disease [16,17,18]. The zoonotic potential of BLV was recently documented by the presence of retrotranscribed BLV DNA in the breast tissue of women suffering from breast cancer and by a case–control study that shows that some breast cancer cases were associated with BLV exposure [19,20]. These data provide an indication that BLV cannot be restricted to lymphoma in cattle and could also be a public health concern.

According to the OIE Terrestrial Animal Health Code, EBL is a notifiable disease and official control measures must be carried out, including monitoring, precautions at borders, control of movement inside the country, and slaughtering of BLV-infected cattle at government expense. The agar gel immunodiffusion test (AGID) and enzyme-linked immunosorbent assay (ELISA) are used as routine diagnostic tests for serological screening of BLV infection; however, ELISA due to its high sensitivity and specificity is the prescribed test for trade [21]. In most European countries, national eradication programs, based on the test-and-slaughter approach, have been successfully applied to achieve BLV-free status [22,23,24]. However, many other countries from different continents are not able to implement such programs successfully because of the high prevalence among cattle and the lack of financial support from the authorities toward owners of infected animals [25].

Pakistan has a large livestock population, represented by the species within the *Bovidae* subfamily, currently counting close to 50 million cattle and 40 million water buffalo [26]. Both dairy cattle and water buffalo are important species in the rural economy in Pakistan as a part of the livestock population used as the main source for dairy production and as draught animals. In the past, some serological studies were undertaken, providing proof for the presence of BLV infection in dairy cattle and water buffalo [27,28]. However, a large serological survey for infection with BLV has not been performed thus far. The objective of this study was to estimate the true seropositivity to BLV at the farm and within-farm levels in Pakistan, using a latent class analysis. In addition, some factors potentially influencing BLV seropositivity were analyzed.

## 2. Materials and Methods

### 2.1. Study Population and Sample Collection

According to the Economic Survey of Pakistan [26], there are 47.8 million cattle in Pakistan. Originally, we planned to perform stratified random sampling from all seven regions, taking into account the proportional allocation of cattle within each region. In this study, number of animals was determined using the sample size estimation formula, n = Z^2^pq/L^2^ [29], where n is the sample size, Z is the normal deviate (1,96) at 5% level of significance, p is 20% of expected prevalence on the basis of a previous study [28], q is the 1-p, and L is the acceptable error (2%) of prevalence. This gives us a value 1537 animals. However, due to practical limitations, mainly the difficulties of gaining access to farms, especially the small farms in the mountain regions of the country, it was impossible to fulfil these initial objectives, and in fact we had to make a correction in the sampling plan. Finally, blood was taken from a total of 1380 dairy cattle, which accounted for 0.0028% of the total population of cattle in Pakistan, selected from 451 farms (0.0040% of the total number of recorded farms) located in 60 different districts of seven regions of Pakistan. A complete description of the cattle population, number of herds, number of samples tested, and respective number of tested herds located in all regions is given in Table 1. The selected farms were categorized into three groups, defined as farms that included the following number of cattle: 65 farms equal to or greater than 100 animals, 37 between 99 and 50 animals, and 349 farms with fewer than 49 cows. In general, the sampling scheme mainly took into consideration the regions with predominant cattle production in the country, i.e., the Khyber Pakhtunkhwa, Punjab, and Sindh regions [26,30]. The sampling was conducted during the period from March 2017 to April 2018 from seven regions of Pakistan: Punjab, Khyber Pakhtunkhwa, Sindh, Gilgit Baltistan, Balochistan, Islamabad Capital Territory, and Azad Jammu and Kashmir. Additionally, blood was taken from 92 water buffalo, wherein 50 came from 21 herds where cattle and buffalo were housed together and 42 were located in 8 herds housing only water buffalo. All sampled animals were at least 2 years old. The majority of the cattle belonged to an indigenous breed such as Sahiwal, Red Sindhi, Cholistani, Dhanni, Bhagnari, Djal, Lohani, Rojhan, Achay, or Kankrej, with the predominance of Red Sindhi and Sahiwal and also exotic breeds (Holstein, Friesian, Jersey). Additionally, some sampled animals represented crossbreeding of indigenous and exotic breeds. The sampled buffaloes were riverine-type and mainly belonged to four breeds, i.e., Nili-Ravi, Kundi, Rovi Bori, and Badine. Along with the sampling procedure, a computerized list of animal identities was created and a standardized questionnaire about the farm location, their size, animal species, and breed was also prepared.

### 2.2. Ethical Statement

Sampling was carried out by veterinarians, and the collection of blood samples was done under the permission of the farm owner and according to the permit of COMSATS Institute of Information Technology, Islamabad, no. CIIT/Bio/ERB/17/26.

### 2.3. Detection of Antibodies

A 10 mL blood sample from each animal was collected from the jugular vein, and the samples were allowed to clot and then transferred onto ice as quickly as possible to the laboratory of the National Veterinary Laboratories in Islamabad. Next, the sera were collected, frozen at −20 °C, and sent to the OIE Reference Laboratory at the National Veterinary Research Institute in Pulawy, Poland, for serological testing. The sera were evaluated for anti-BLV antibodies using an ELISA kit (IDEXX Leukosis Serum X2 Ab Test, IDEXX, Liebefeld-Bern, Switzerland). All sera that were positive by screening test were retested by an IDEXX Leukosis Blocking Ab Test (IDEXX, Montpellier, France), which was used as a confirmatory assay. All test procedures were applied according to the manufactures’ instructions and the plates were read on an ELISA reader (Multiskan FC, Thermo Scientific, Ratastie, Finland).

### 2.4. Statistical Analysis

The apparent and true prevalence of infection with BLV at both the animal and farm levels was estimated using a statistical model based on the Bayesian approach [31]. The apparent animal prevalence was calculated as the number of test-positive animals among the total number of animals tested, while the apparent farm prevalence was calculated as the number of test-positive farms among the total number of farms tested. A farm was considered positive when at least one animal showed the presence of antibodies in the ELISA test. In this study, we used beta-binomial models to estimate both the animal and herd prevalence according to a commonly accepted formula [31,32,33,34]. Prior estimation of the sensitivity and specificity of ELISA, on the basis of available data from test’s manufacturer, was 99.6% (95% CI: 98.4–99.9) and 100% (95% CI: 98.8–100), respectively. The analysis was conducted by the Bayesian approach using WinBUGS [35]. For model analysis, the OpenBUGS 3.2.2.2 software was used, which includes a suitable Markov chain Monte Carlo (MCMC) scheme based on Gibbs sampling. The ELISA data were parameterized using the R free software environment for statistical computing. The true prevalence (TP) was calculated as the median and 95% posterior credibility intervals. In order to identify risk factors potentially associated with seropositivity to BLV infections at the herd level, we developed a statistical model and calculated the odds ratio (OR) on the basis of logistic regression models using the Bayesian approach [36]. The Bayes approach was used to take into account the uncertainties resulting from the small number of positive herds detected in this study and the large unevenness of particular groups of animals that were considered in relation to the risk factor characteristics. Statistical calculations were performed using the R free software environment with the *rbeta* function.

## 3. Results

In total, 1380 cattle and 92 water buffalo serum samples were tested by ELISA. A total of 52 (3.77%) cattle sera were positive and seven (1.55%) herds with at least one seropositive animal were detected (Table 2). All sera that were positive by the IDEXX Leukosis Serum X2 Ab Test were also positive by the IDEXX Leukosis Blocking Ab Test, used as a confirmatory assay. Out of seven regions, seropositive cattle were found in five regions, with the highest seroprevalence in Punjab (8.82%) and Khyber Pakhtunkhwa (5.88%), excluding Islamabad Capital Territory, and Azad Jammu and Kashmir where no positive results were recorded. All 92 serum samples from water buffalo were identified as negative.

Table 2 also shows the overall seroprevalence estimates of the posterior median and 95% credibility intervals. Analysis at the within-herd and herd levels showed that 3.8% (95% CI: 2.8–4.8) of animals and 1.4% (95% CI: 0.7–3.1) of herds were truly seropositive. By inclusion of the regions as a covariant in the model, it was possible to estimate the seroprevalence per region. The estimated medians for the within-herd true seroprevalence were 5.9% (95% CI: 3.9–8.6) and 8.5% (95% CI: 5.9–12.7) for the Khyber Pakhtunkhwa and Punjab regions, respectively. Lower values were found in the regions of Sindh 0.5% (95% CI: 0.1–2.0), Balochistan 1.9% (95% CI: 0.6–5.3), and Gilgit Baltistan 1.0% (95% CI: 0.2–5.7). The respective 95% credibility intervals overlapped, showing virtually only one distribution for all of these regions. In the Islamabad Capital Territory and Azad Jammu and Kashmir regions, however, where no BLV infections were noted due to the statistically low number of animals tested, we could not exclude the occurrence of BLV infection at the low range of seroprevalence. In these regions, the posterior estimates for the median of the within-herd level true positive cattle were less than 0.3% (95% CI: 0.0–4.2). The true seroprevalence at the herd level varied from 0.9% (95% CI: 0.0–15.1) to 2.7% (95% CI: 0.5–12.7), and the within-herd level was similar with the respective 95% credibility intervals within ranges (minimum and maximum values) that were very similar to each other.

Various factors that could hypothetically be associated with seropositivity to BLV were evaluated (Table 3). When the herd size of cattle was taken into account, medium herds (50) had a significant effect (OR = 23.57, 95% CI: 3.01–103.48) on increased BLV seropositivity over large herds (≥100) (OR = 8.09, 95% CI: 3.08–65.82). Despite this fact, no statistically significant association between herd size and within farm seroprevalence was noted. The analysis also demonstrated that common housing of cattle representing indigenous breeds with exotic breed cattle (OR = 0.67, 95% CI: 0.06–2.35) or housing indigenous or their crossbred cattle with exotic breed cattle (OR = 0.95, 95% CI: 0.14–3.01) or with their crossbred cattle (OR = 1.30, 95% CI: 0.20–4.21) had no significant effect on the BLV seroprevalence. Common housing of water buffalo and cattle seems to not be a risk factor contributing to an increased BLV seropositivity since the high median value (OR = 27.10, 95% CI: 0.63–119.34) was the result of an occurrence of BLV infections in herds that kept cattle only.

## 4. Discussion

Seroepidemiological surveys have showed that infections with BLV are widespread in many countries around the world [37]. In this study, we estimated the true seroprevalence of infection with bovine leukemia virus in Pakistan at the animal and herd levels. According to the author’s knowledge, this is the first large serological survey on BLV infection in the cattle population in Pakistan. Sampled animals were tested by ELISA, and then the obtained data on apparent prevalence were incorporated into a statistical model by the use of Bayesian inference, as was recently described by Olech et al. [38]. We used this analysis method since the sampling system used in this study did not reflect the cattle population in the analyzed areas of the country with accepted accuracy and was subjected to some level of uncertainty caused by the lack of herd stratification, different herd sizes, and different numbers of samples collected from particular herds. This was due to some limitations of the sampling strategy, and despite the initial plan to perform random sampling, we had to resort to a more convenience sample collection.

The median posterior estimate of the herd level true BLV prevalence was 1.4%, with a 95% credible interval 0.7–3.1; however, some differences were noted between regions. Since their respective 95% credibility intervals were overlapped and closely related to each other, we cannot postulate with 95% certainty that there was a significant association between the BLV seroprevalence and the studied regions. These observations are consistent with other studies, indicating a rather stable nature of BLV prevalence in endemic areas, as was reported in Egypt [39] and the USA [40]. This does not, of course, preclude the need for further studies involving more herds and more animals, as well as analyses of herd-specific risk factors that may favor inter-herd BLV transmission. The median posterior estimate for the within-farm true seroprevalence was 3.8% with a 95% credible interval of 2.8–4.8. The median prevalence rate was considerably lower than those reported in other Middle East countries, i.e., 25% in Iran [41], 8% in Iraq [42], and 25% in the United Arab Emirates [43]. It was also not consistent with the seroprevalence reported previously in Pakistan. Earlier studies used the agar gel immunodiffusion test (AGID), and a limited number of cattle showed no seroreactivity to BLV [27]. However, a recent study carried out with the use of ELISA (IDEXX Leukosis Serum X2, IDEXX, Liebefeld, Switzerland) reported 20% seroreactivity when 600 cattle from herds located in the Khyber Pakhtunkhwa region were tested [28]. In the present study, the median true prevalence in this region was 5.9% with a 95% credible interval of 3.9–8.6. The above-mentioned differences in seroprevalence can probably be attributed to the different sensitivities and specificities of the serological tests used. It is well known that one of the limitations of AGID is its lower sensitivity compared to ELISA [44]. On the other hand, the two ELISA tests used in this study, representing different variants of this technique, i.e., indirect and blocking ELISA, are highly reliable due to their very high sensitivity and specificity [45]. Therefore, we believe that the effect of potential misclassification of samples in this study will be minimal. Moreover, the use of Bayesian analysis provided a possibility for elimination of misclassified animals. 

In order to identify risk factors potentially associated with seropositivity to BLV infections in Pakistan, we developed a statistical model and calculated odds ratios (OR) with 95% confidence intervals on the basis of logistic regression models and the Bayesian approach. The OR values lower than 1.0 testified to the lack of association between the factors involved in the statistical analysis, and higher than 1.0 demonstrated risk factors favoring BLV seropositivity. Herd size was highlighted as a key factor that had a significant effect on the increased risk for BLV infection in cattle [39,41,43,46]. In Egypt, herds with large sizes (>200 and >300) resulted in a seroprevalence higher than 24% and 35%, respectively, while smaller herd sizes (<50) registered a seroprevalence of 12% [39]. A similar finding was made in Iran where the seroprevalence in herds with more than 250 cattle was approximately two times higher than in herds accommodating less than 100 animals [41]. In this study, when the herd size was initially expressed as a large herd (≥100 cattle), the impact of increasing the risk of BLV infection in large herds was confirmed with the median value OR = 8.09. This tendency was also seen when the herd size was reclassified and expressed as a medium herd (50–100 cattle), which indicated that, in Pakistan, the definition of critical herd size predisposing to BLV infection is related to herds counting at least 50 cows. In turn, this leads to the conclusion that in herds with less than 50 heads, the risk of BLV infection is negligible. The statistical significance of large- or medium-sized herds being associated with seropositivity to BLV can be explained in the context of the different husbandry and farm management systems in use for the analyzed herds. Since BLV is mostly transmitted horizontally [47], it is logical that high livestock density allows for easy direct contact between cattle, which was reported as a risk factor promoting BLV seropositivity [37,48]. 

The introduction of cows without knowledge of their infection status to the herds is the most significant risk factor contributing to an increased spread of BLV infection [7,49]. Even though the purchasing of cattle from other herds in the country or from other countries was not evaluated in this study as a risk factor, its role in the transmission of BLV is obvious [8]. During the last two decades, there have been significant changes in dairy cattle breeding in Pakistan due to a rapidly growing demand for milk and dairy products and also due to the development of milk processing industries. Industrial or semi-industrial dairy farms, which are an important part of the dairy industry in Pakistan, are mainly located in Punjab region, which has the largest cattle population, with 48% of the total dairy cattle population, followed by Sindh with 23%, KPK with 20%, and Baluchistan with 7% [26]. Therefore, the higher BLV seroprevalence noted primarily in Punjab and Khyber Pakhtunkhwa could be an effect of a high stocking density of animals in herds located in these regions, or it could be explained by extensive import of live animals from other countries, mainly the USA, where infection with BLV was noted [40]. In fact, almost all herds enrolled in this study had introduced cattle from other farms from Pakistan or from abroad. In addition, the uncontrolled exchange of adult breeding males between farms and the purchasing of semen straws, which is practiced on many farms, pose a risk of exacerbating BLV infections when biosecurity measures are ignored [50,51].

One typical characteristic of dairy cattle breeding in Pakistan is farms housing indigenous breeds together with exotic as well as their crossbreed cattle. All of Pakistan’s indigenous cattle are Zebu (humped type, *Bos indicus*), and there are 15 recognized breeds in the country, including Red Sindhi and Sahiwal as the dominant breeds. This breeding system is widely used throughout the country and there are a large amount of initiatives of industrial farms as well as smallholder farmers to use of exotic dairy cattle, mainly Holstein, Frisian, and Jersey, in order to adopt their genetic potential. The rationale behind the crossing of indigenous with exotic breeds is that the productivity of dairy cattle crossbreeds is far higher than that of indigenous breeds, with longer lactation time, higher milk production, and shorter calving intervals. These advantages make crossbred cattle highly preferred for intensive and semi-intensive dairy farming systems [30]. The population of crossbreed cattle is continually increasing, and represents about 13% of Pakistan’s total cattle population, while purebreds account for 43%, and nondescript for 44% [28]. We considered that the common housing of different breeds could be a factor promoting transmission of BLV. The rationale for that was that Jersey and Holstein cattle are breeds highly susceptible to BLV [52] and can be a source of virus diffusion following contact with indigenous cattle or their crossbreeds. The present study took into account indigenous breeds, mainly Red Sindhai and Sahiwal as well as Thari and Bagh Nari and exotic Friesian and Jersey breeds. It was demonstrated that neither the common housing of indigenous cattle with exotic breed animals (OR = 0.67) nor the common housing of indigenous or their crossbreed cattle with exotic breed animals (OR = 0.95) or with their crossbreeds (OR = 1.30) had a significant effect on BLV seroprevalence. However, it is recommended that this study be extended over other indigenous breeds and crossbreeds in the context of their susceptibility to BLV infection. A recent study showed that the prevalence of BLV infection and the proviral loads among native cattle from Myanmar was significantly lower than those observed in crossbred Holstein Friesian/native cattle, suggesting lower susceptibility of the native breed to BLV [53]. In addition, some studies revealed that the susceptibility to BLV infection in different cattle breeds, including indigenous cattle, is associated with a polymorphism of the BoLA-DRB3 gene [54,55,56]. 

Buffaloes are one of the major milk-producing animals in Pakistan, representing about 48 percent of the total dairy herd and providing 62 percent of total milk production [26]. In the present study, all 92 serum samples from water buffalo were negative, as was noted in other studies showing a lack of positive results when animals from Taiwan [57], Cambodia [58], and Brazil [59] were tested. In contrast, seropositivity to BLV was noted at 4.3% in Brazil [60], 2% in Venezuela [61], and at 9% in Egypt [62]. When the main uncertainty factor, being the low number of sampled animals, was taken into account, Bayesian inference allowed for the predictive range of BLV prevalence to be estimated at a level not higher than 0.2%, with a 95% credible interval of 0.0–3.2. These results may indeed indicate the possible occurrence of BLV-seropositive buffalo in Pakistan at a low level. This would be in agreement with earlier studies showing 0.8% seropositivity in water buffalo in Pakistan [27]. Assuming that buffalo in Pakistan may show a low level of BLV seropositivity, its exact estimation requires a higher number of animals to be tested. On the basis of the high median value (OR = 27.10), one can suspect that in herds housing water buffalo together with cattle, there is minimal risk of BLV infection as compared to herds maintaining cattle alone. Thus, the common housing of water buffalo and cattle does not appear to be a risk factor promoting BLV infection. 

## 5. Conclusions

In conclusion, the present study provides evidence that BLV infection in Pakistan is endemic. Further studies are required to establish effective prevention and control measures. These studies should be oriented towards the acquisition of new knowledge about epidemiological dynamics of BLV infection, especially in large- and medium-sized herds. In parallel, risk factors, other than evaluated in the present study, favoring inter and within-herd BLV transmission such as insect density population, farming procedures, and dairy cattle trading should be elucidated. This will help to establish appropriate measures that will prevent BLV transmission in dairy herds in Pakistan.

## Figures and Tables

**Table 1:** Overview of the sampling scheme used in this study

Region	Cattle Population in Region	Number of Sampled Cows	Percentage of Tested Cows	Estimated Number of Herds of Cows	Number of Sampled Herds of Cows	Percentage of Tested Herds
Khyber Pakhtunkhwa	11,528,313	374	0.0032%	2,603,437	139	0.0053%
Punjab	25,544,987	272	0.0011%	6,467,939	49	0.0008%
Sindh	9,670,872	348	0.0036%	1,630,781	121	0.0074%
Balochistan	2,874,416	160	0.0056%	340,105	40	0.0118%
Gilgit Baltistan	681,909	91	0.0133%	101,795	76	0.0747%
Islamabad Capital Territory	82,805	70	0.0845%	11,593	9	0.0776%
Azad Jammu and Kashmir	562,903	65	0.0115%	112,580	17	0.0151%
Total	50,946,205	1380	0.0027%	11,268,230	451	0.0040%

**Table 2:** Within-farm and farm-level seroprevalence estimates of BLV infection per region, summarized by the median and 95% credibility intervals.

Region	Within-Farm Apparent Seroprevalence	True	Farm-Level Apparent Seroprevalence	True
Khyber Pakhtunkhwa	22 (5.88%)	5.9% (3.9–8.6)	2 (1.44%)	1.4% (0.3–5.0)
Punjab	24 (8.82%)	8.5% (5.9–12.7)	1 (2.04%)	2.2% (0.4–10.8)
Sindh	2 (0.57%)	0.5% (0.1–2.0)	2 (1.65%)	1.5% (0.4–5.8)
Balochistan	3 (1.88%)	1.9% (0.6–5.3)	1 (2.50%)	2.7% (0.5–12.7)
Gilgit Baltistan	1 (1.10%)	1.0% (0.2–5.7)	1 (1.32%)	1.6% (0.0–26.0)
Islamabad Capital Territory	0 (0.00%)	0.3% (0.0–4.2)	0 (0.00%)	1.6% (0.0–26.0)
Azad Jammu and Kashmir	0 (0.00%)	0.3% (0.0–4.7)	0 (0.00%)	0.9% (0.0–15.1)
Total	52 (3.77%)	3.8% (2.8–4.8)	7 (1.55%)	1.4% (0.7–3.1)

**Table 3:** Risk factors associated with seropositivity to BLV infection in Pakistan.

Risk Factor	Categories	Number of Farms		OR (CI 95%)
		BLV+	BLV-	
Large herd	≥100	5	60	8.09 (3.08–65.82)
	≤99	2	384	
Medium herd	≥50	6	96	23.57 (3.01–103.48)
	≤49	1	348	
Farm with different breeds	Indigenous breed	1	84	0.67 (0.06–2.35)
	Exotic breeds	5	137	
Farm with different breeds	Indigenous or their crossbreed	2	138	0.95 (0.14–3.01)
	Exotic	5	211	
Farm with different breeds	Indigenous or their crossbreed	2	138	1.30 (0.20–4.21)
	Exotic or their crossbreed	5	297	
Cohabitation of water buffalo and cattle	Cattle	7	317	27.10 (0.63–119.34)
	Cattle and water buffalo	0	127	

## Data Availability

The data presented in this study are available on request from the corresponding author.
